# The Use of Wind Turbine Blades to Build Road Noise Barriers as an Example of a Circular Economy Model

**DOI:** 10.3390/ma17092048

**Published:** 2024-04-26

**Authors:** Mirosław Broniewicz, Anna Halicka, Lidia Buda-Ożóg, Filip Broniewicz, Damian Nykiel, Łukasz Jabłoński

**Affiliations:** 1 Faculty of Civil Engineering and Environmental Sciences, Bialystok University of Technology, 15-351 Białystok, Poland; filip.broniewicz@pb.edu.pl; 2 Faculty of Civil Engineering and Architecture, Lublin University of Technology, 20-618 Lublin, Poland; a.halicka@pollub.pl (A.H.);; 3 The Faculty of Civil and Environmental Engineering and Architecture, Rzeszow University of Technology, 35-959 Rzeszów, Poland; lida@prz.edu.pl (L.B.-O.); d.nykiel@prz.edu.pl (D.N.)

**Keywords:** composite structures, turbine blades, acoustic screens, FEM analysis, structural behavior, saving energy

## Abstract

This project’s objective was to create a circular economy in the composites sector by examining the possibility of using wind turbine blade composite materials to construct noise-absorbing barriers for roads. The possibility of constructing road noise barrier panels from components obtained from turbine blades was conceptually examined, and the geometry and construction of wind turbine blades were evaluated for their suitability as filler components for panels. The tensile strength parameters of two types of composites made from windmill blades—a solid composite and a sandwich type—were established based on material tests. The strength of the composite elements cut from a windmill propeller was analyzed, and a three-dimensional numerical model was created using the finite element method. The strength values of the composite used to construct the noise barriers were compared with the stresses resulting from loads operating on the road noise barriers, as determined in compliance with current standards. It was discovered that acoustic screens composed of composite materials derived from wind turbine blades may withstand loads associated with wind pressure and vehicle traffic with sufficient resistance. In order to evaluate the environmental benefits resulting from the use of composite material made from wind turbine blades to make noise barriers, this study presents the values of the embodied energy and embodied carbon for several types of road noise barriers using life cycle assessment.

## 1. Introduction

A strategy for attaining sustainable development that has grown in favor among companies, legislators, and economists is the circular economy. Although there are many different circular economy concepts, they all present a novel way to increase value and eventually prosperity by extending the life of products and moving waste from the end of the supply chain to the beginning, which results in multiple efficient resource uses [1,2]. The current manufacturing process includes extracting natural resources from the environment, converting them into new materials, and then returning the new materials to the environment. There is a beginning and an end to this sequential process. This system’s limited resources will soon run out. Waste accumulates and can lead to contamination or expensive disposal. Furthermore, industrial processes are often inefficient, wasting more resources in the process. On the other hand, a circular economy makes new products out of leftover materials. Everything is reused, recycled whenever feasible, or in the worst-case scenario, converted into raw materials or used as a source of energy.

**Figure 1:**
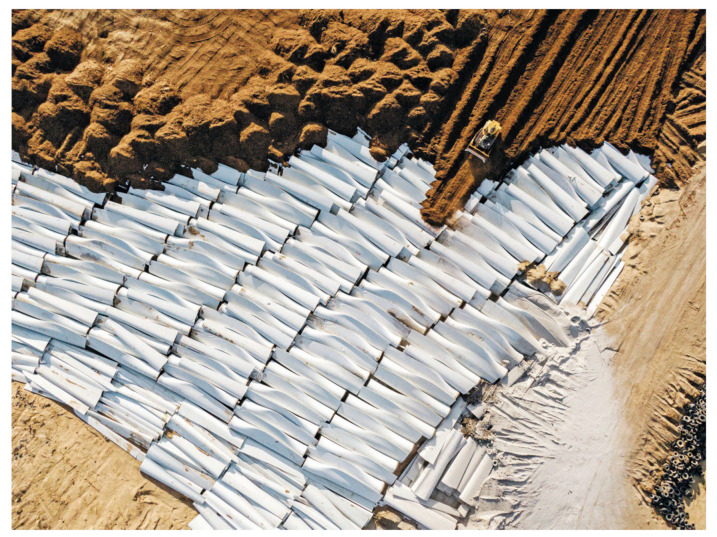
Wind turbine blades buried in Casper, Wyoming (Benjamin Rasmusen) [3].

The topic of what happens to the waste materials from the first commercial wind turbines, which went online in the mid-to-late 1990s, arises as they approach the end of their operating lifespan and are ready to be decommissioned. There is a misconception that because certain parts of wind turbines, such as their blades, cannot be recycled, these turbines negatively affect the environment (Figure 1) [3]. Although it is true that some products cannot be recycled, producers and owners go to tremendous lengths to make sure they are as sustainable as possible in the end by looking for ways to recycle, reuse, or rebuild them using recyclable components in the future. A wind turbine is composed of recyclable materials to a degree of about 96%. Steel, copper, aluminum, other precious metals, recyclable polymers, and other materials are commonly used to make their outer shell, shafts, gearing, and electrical components. The majority of the materials used to make the blades are fiberglass. An onshore wind turbine’s average blade length is about 50 m. Nonetheless, there is an increasing tendency toward higher turbines, which are frequently located offshore at sea and have blade spans as long as 80–90 m. Fiberglass cannot be recycled entirely. Upon recycling, it becomes much more challenging to process the composite material consisting of tiny plastic and glass strands, making it non-biodegradable. Usually though, it is burned or dumped as trash in landfills. Not every first-generation commercial blade is intended for landfills, even though the majority of them are handled as waste. Its basic materials are recycled in a variety of creative ways so they can be used again in new constructions or as raw materials for future buildings [4].

Governments are encouraging, and in some cases mandating, the adoption of circular economy principles, which would lead to increased resource efficiency and decreased waste [5]. As implemented into the economies of many countries, the basic principles of the circular economy involve producing products that use fewer primary resources, last longer, and can be repaired, recycled, or used for other purposes when they reach the end of their useful life.

As part of the research project, a concept for creating highway noise barriers out of composite material made from wind turbine blades will be developed. The following phases will be included in the research project:

1. Performing conceptual analyses of the application of wind turbine blade composite elements for building noise barriers on roads;
2. Performing material tests for blades by obtaining samples for testing and performing material tests on the laminates (tension, compression, and modulus of elasticity);
3. Development of a preliminary FEM model, taking into account the results of material testing;
4. Performing panel strength calculations in compliance with relevant standards through assessment of the acoustic panels’ strength, durability, and safety properties;
5. Experimental tests of a natural-scale panel;
6. Assessment of the sound-absorbing properties of the acoustic panels by conducting acoustic tests, determining the acoustic parameters, and modeling using software tools;
7. Validation of the numerical model using research from experiments to investigate how the panels’ most crucial technical parameters affect their ability to support loads and their acoustic qualities (parametric analysis will be used);
8. Performing a life cycle analysis for acoustic panels made of composite elements obtained from wind turbine blades.

This article presents the results of the first four stages of the entire research task, covering the first phase of the research project. As part of this stage of the research, a supporting structure as well as a replaceable acoustic panel made of composite material were proposed, whose dimensions were adapted to the typical acoustic screens currently used. Then, a conceptual analysis was conducted for how to cut fragments of wind turbine blades into components that can be easily used to build a screen, taking into account both the complex geometric shape of the blades and the various types of materials used. In the next stage, an FE numerical model of an acoustic panel made of materials taken from used wind farm blades was built, and the load-bearing capacity of the screen for standard loads was tested analytically and numerically. In the next part of the work, the screen will be experimentally tested at a natural scale on a test stand in order to validate the numerical model and examine the acoustic properties of the screens “in situ” and in a reverberation room.

## 2. Geometry and Construction of a Noise Barrier

A road noise barrier is an artificial barrier that prevents sound waves from traveling from the source of the noise, which is traffic noise, to the region next to the road, which is sound-free. The effectiveness of a road screen is determined by the amount of noise that is absorbed by the screen and the amount that is transported to the acoustic shadow zone due to wave refraction [6].

**Figure 2:**
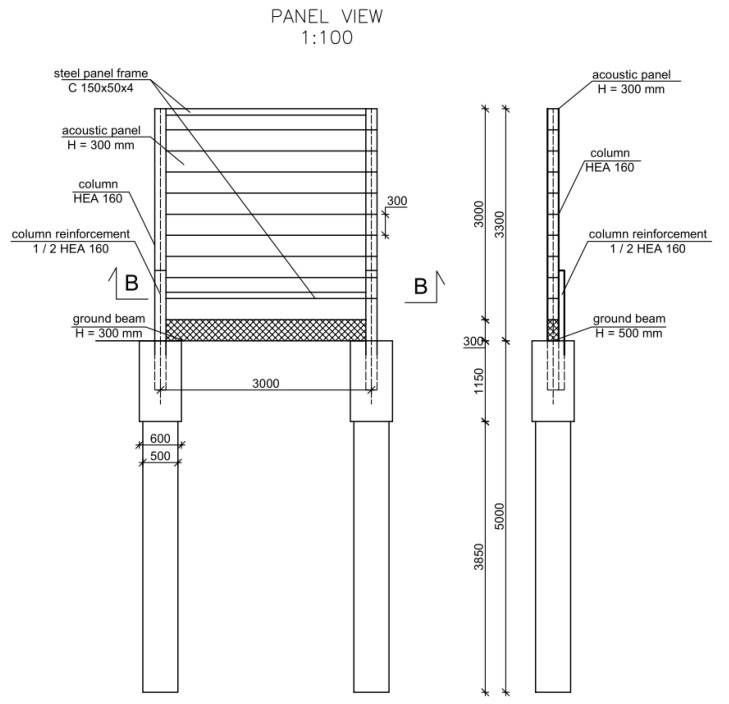
Geometry of the road noise barrier.

Regardless of the materials used in their construction, road noise protection devices can be categorized based on their mechanical characteristics under normal operating conditions [7,8]. Design standards also set the parameters for assessing the safety and impact of devices on the environment [9,10]. Preventing permanent deformation, irreversible displacement of acoustic elements, separation of individual panels, and detachment from fasteners or supports is also crucial. The case study involves an acoustic barrier consisting of steel columns on a pile foundation, a ground beam, and composite acoustic panels. The 3.0 m × 3.0 m composite acoustic panel consisted of 10 elements 3000 mm long, 300 mm wide, and 30 mm thick cut from wind turbine blades, which were connected to each other on both sides and longitudinally with 3M VHB tapes. The entire panel was mounted in a frame made of cold-formed U sections measuring 100 × 50 × 4 mm and stabilized with spacers or rivet nuts. The geometry and cross-section of the tested acoustic screens are shown in Figure 2.

The panel being presented was engineered to achieve a high sound dispersion coefficient while adhering to mechanical specifications that allowed it to be used in standard road noise barrier structures. The sandwich construction of the screen, with a balsa wood filler sandwiched between two composite layers, shapes its acoustic absorption regardless of the effect of sound dispersion. The structure is double-sided and can be used interchangeably, allowing various configurations in the screen structure.

## 3. Propeller Laminates and Their Mechanical Characteristics

**Figure 3:**
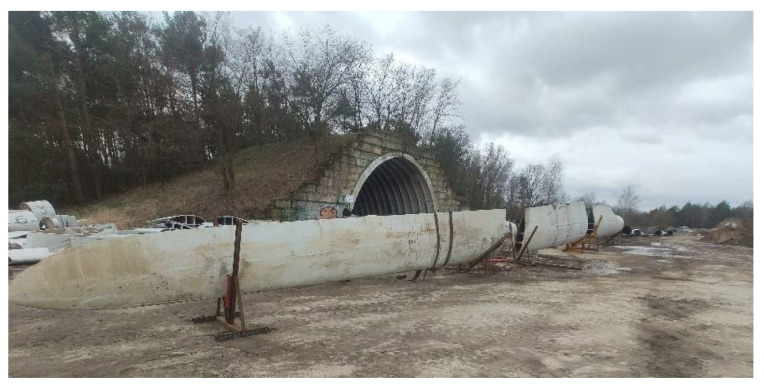
Wind turbine blade.

**Figure 4:**
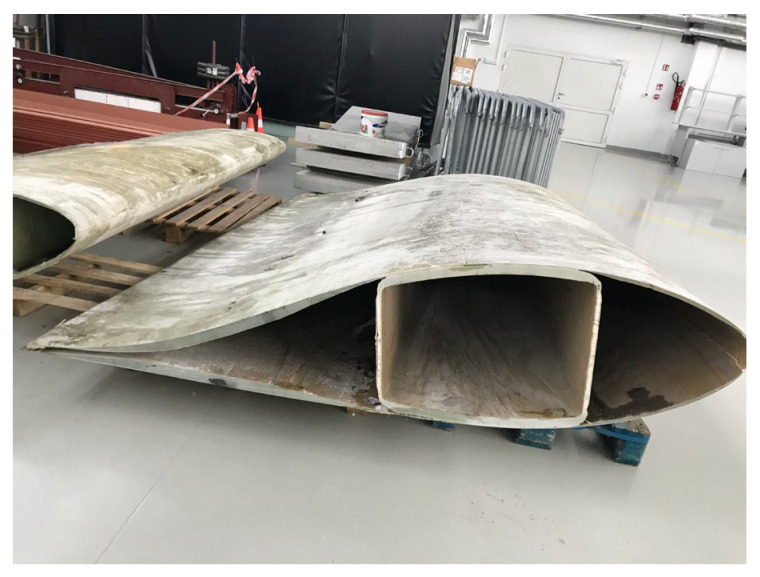
Sections cut from wind turbine blade.

A wind power plant blade measuring 36 m was used to create the composite elements of the road noise barrier panel (Figure 3) [11]. From the middle of the blade’s length, two three-meter-long segments were cut (Figure 4).

**Figure 5:**
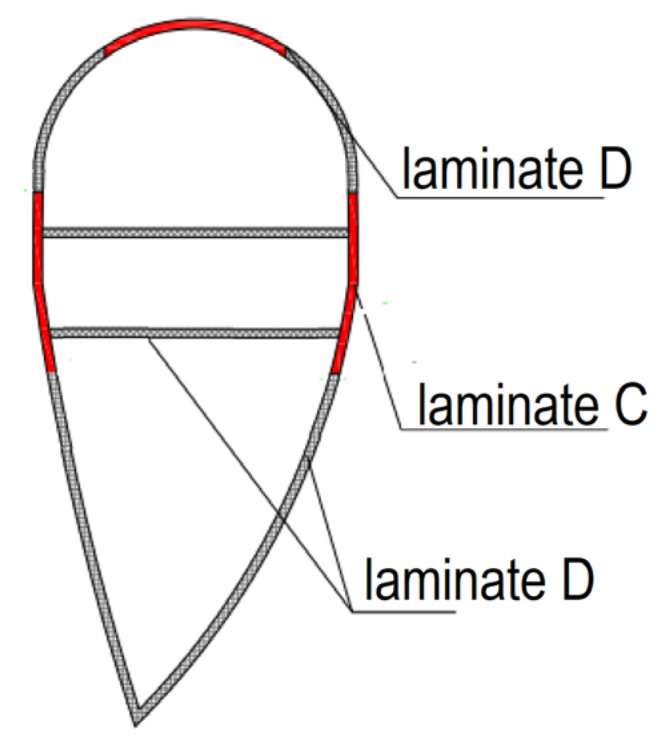
Types of laminates used in the construction of windmill blades.

Of the four types of laminates found in wind turbine blades, only two can be used to create acoustic barriers. Laminate C is composed entirely of fibrous composite layers. Laminate D is a sandwich material consisting of an inner layer (core) made of balsa wood and outer layers consisting of a composite of fiberglass and resin (Figure 5).

Determining the values of the partial safety coefficients and the material coefficients for composite elements to establish the design values of the mechanical properties requires more effort than for standard materials. The variety of composite materials, variations in the uncertainties in models predicting the element’s strength for a particular kind of damage, and the numerous mechanical characteristics of the composite material all have an impact on these variables [11,12,13]:

(1) $$R_{d}=\frac{1}{\gamma_{Rd\;}\cdot {\;\gamma }_{M}}\cdot \eta_{c,i}\cdot R_{k,i}$$

where $R_{d}$ is the design values of the material properties, $\gamma_{Rd}$ is the partial factor to account for uncertainties in the model, $\gamma_{M}$ is the partial factor to account for unfavorable deviations in the material property values from their characteristic values, $\eta_{c,i}$ is the total conversion factor, taking into account the effects of moisture, temperature, and material ageing, and $R_{k,i}$ is the characteristic values of the material properties.

The values of each type of laminate’s mechanical and physical properties are shown in Table 1 and Table 2 presents the configurations of the individual layers.

## 4. Numerical Model of the Acoustic Composite Panel

Finite element modeling of a curved composite element forming the barrier’s panel was performed using ANSYS 2022 software. The laminate C and D elements were verified. To verify the elements’ resistance to wind and traffic loads, numerical models of the elements were prepared. The geometry of the models was based on the actual dimensions of the tested elements. Tests performed on the composite’s mechanical properties provided the basis for the material’s properties.

**Figure 6:**
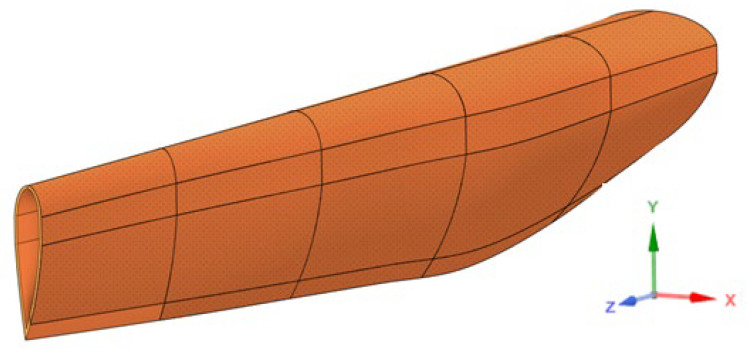
**A** 3D model of the blade.

**Figure 7:**
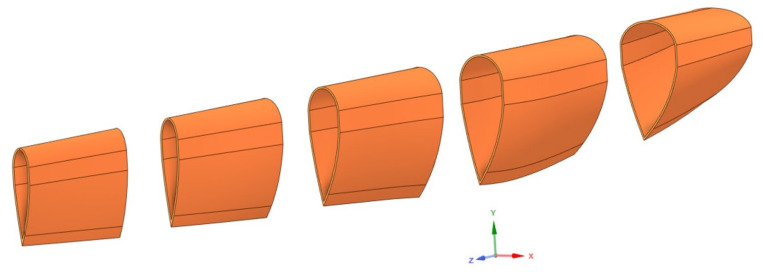
Division of the part into three-meter-long segments.

**Figure 8:**
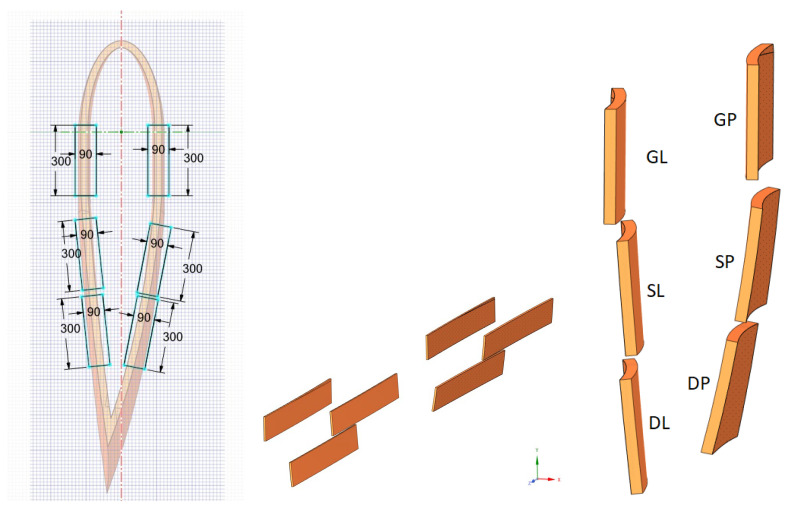
Components of panels cut from windmill blades. GL = left side solid element; GP = right side solid element; SL(DL) = left side sandwich element; SP(DP) = right side sandwich element.

Figure 6 shows the 3D model of a windmill blade fragment made with ANSYS Spaceclaim 2022 software [15]. Beginning at the point where the blade connected to the propeller axis, this fragment spanned the first 17 m of the blade’s length. To create the acoustic screen, the model was divided into sections that were 3 m in length (Figure 7). These pieces should be arranged within a channel frame and fastened to HEA160 steel columns. Subsequently, composite plate elements measuring 3000 mm in length and 300 mm in width were cut from each segment with the purpose of using them as noise barrier panel elements (Figure 8). The wall thickness of these elements was 30 mm. The geometric measurements, types, and weights of these elements are given in Table 3.

**Figure 9:**
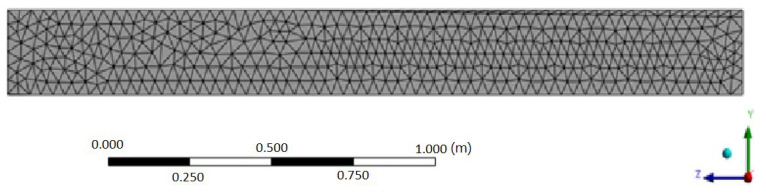
Discretization of the GP element.

The segments were discretized using a spatial mesh and split into solid elements with an element size of roughly 5 cm to perform finite element analysis (FEA) of the panel’s behavio r under loading (Figure 9). All degrees of freedom of the edges of the shorter sides of the elements were restrained in order to replicate the mounting conditions on the acoustic screen panel. The ANSYS ACP module was utilized to model the laminates, considering their fibrous lamina properties and the characteristics of the balsa wood that made up their core layer.

## 5. Verification of the Load-Bearing Capacities of the Composite Elements

The elements that formed the acoustic panel were subjected to loads resulting from vehicle traffic and wind pressure. Initially, the stresses were measured in each of the 30-cm-wide composite strips that made up the panel’s components and subsequently throughout the whole acoustic panel. The self-weight load was determined by assuming the volume weight of the fibrous laminates was 18 kN/m^3^ and the volume weight of the balsa wood was 5 kN/m^3^. The self-weight of the acoustic panels depended on the thickness of the panels, their dimensions, and the arrangement of the filling elements.

The wind load was considered to act perpendicular to the side surface of the wind turbine propeller (X direction). The reference mean velocity pressure value was calculated using the following formula [16]:

(2) $$q_{b,k}=\frac{1}{2}\cdot \rho \cdot v_{b}^{2},$$

where ρ is the density of air, ρ = 1.25 kg/m^3^, and $v_{b}$ is the basic wind speed.

The basic wind velocity is calculated as follows:

(3) $$v_{b}=c_{\mathrm{dir}}\;\cdot c_{\mathrm{season}}\;\cdot v_{b.0}$$

where *c_dir_* is the directional factor, which is assumed to be *c_dir_* = 1.0, and *c_season_* is the season factor, which is assumed to be *c_season_* = 1.0. It was also assumed that the screen was located 150 m above sea level in the II terrain category. A net pressure coefficient $c_{p,net,B}=1.4$ was applied due to the fact that the screens are positioned contiguously:

(4) $$\nu_{b}=1.0\cdot 1.0\cdot 22=22\;m/s\;\nu_{m}\left(z\right)=c_{r}\left(z\right)\cdot c_{0}\left(z\right)\cdot \nu_{b}\;\nu_{m}\left(z\right)=0.81\cdot 1.0\cdot 22\frac{m}{s}=17.8\;m/s$$

The reference mean velocity pressure value is

$$q_{b,k}=\frac{1}{2}\cdot 1.25\cdot 22^{2}=302.5\;N/m^{2}$$

The peak velocity pressure value is

(5) $$q_{p}=\left[1+7\cdot I_{v}\left(z\right)\right]\cdot \frac{1}{2}\cdot \rho \cdot v_{b}^{2}=\left[1+7\cdot 0.244\right]\cdot \frac{1}{2}\cdot 1.25\cdot 17.8^{2}=536\;\;N/m^{2}$$

The wind pressure value is

(6) $${w_{e}=q}_{p}\cdot c_{p,net,B}=536\cdot 1.4=750.4\;N/m^{2}\;$$

The dynamic load due to vehicles was assumed to amount to

$$q_{k}\left(v\right)=800\;N/m^{2},$$

which corresponds to road traffic in the open air at a distance of 3 m from the anti-noise device at a maximum speed of 120 km/h [9].

To calculate the design loads, partial factor values were assumed in accordance with [17]. The load combinations were prepared while following the Eurocode 1 recommendations. The value of the partial factor for dead loads was $\gamma_{M}=1.35$, and for variable loads, it was $\gamma_{M}=1.5$. The design values of the loads are presented in Table 4. The principal stresses in the composite solid panels (GL and GP) and sandwich panels (SL and SP) were measured in directions perpendicular to the panel longer edge (when bending about the Y axis) and parallel to the panel longer edge (when bending about the Z axis).

**Figure 10:**
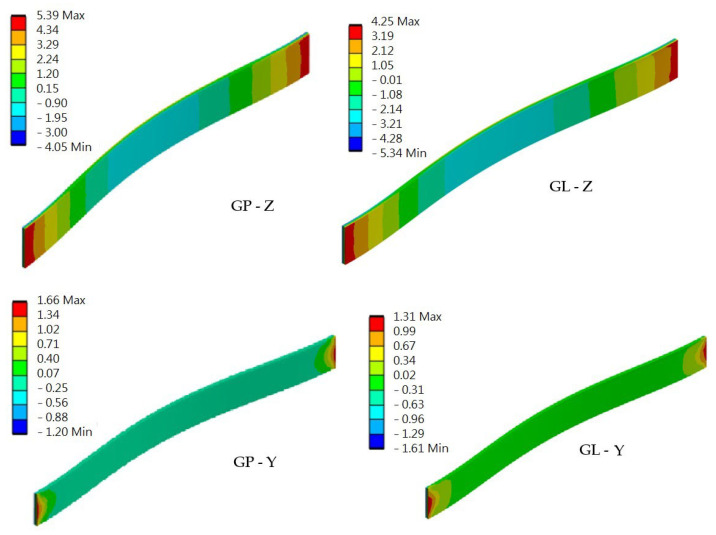
The distribution of stresses in the solid elements GP and GL when bending about the Z and Y axes (MPa).

**Figure 11:**
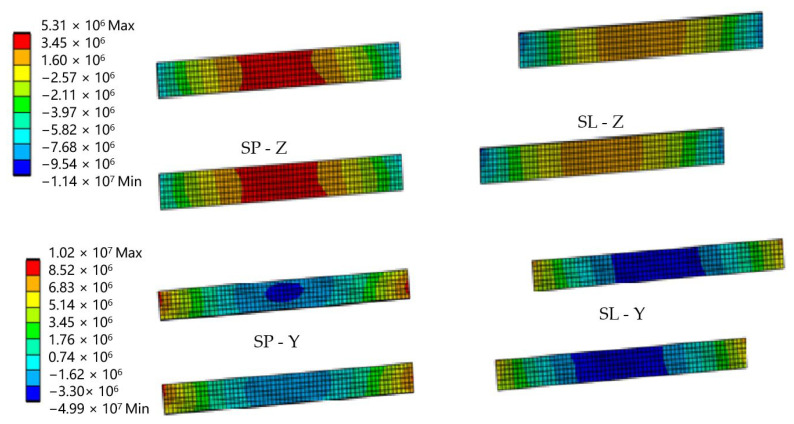
The distribution of the sandwich elements SP and SL when bending about the Z and Y axes (MPa).

Full restraint of the panel ends in the load-bearing columns was assumed. The results were compared with the strength values of the composite material, which are presented in Table 1. The distribution of stresses in solid elements is shown in Figure 10, and the sandwich elements are shown in Figure 11.

A comparison of the maximum compressive and tensile stresses when bending in planes perpendicular and parallel to the surface of the element with the experimentally determined strength is presented in Table 5. The comparison shows that the highest strength utilization occurred when bending an SP element about the Y axis and amounted to 16.8%.

The strength of the composite material used to make acoustic panels was much higher than the stresses caused by wind and traffic loads acting perpendicular to the panel surface, as demonstrated by a comparison of the maximum compressive and tensile stresses when bending in planes perpendicular and parallel to the element’s surface, with the strength determined experimentally. Numerical tests of individual composite elements showed that the maximum tensile stresses that could occur in a solid composite element at 5.4 MPa were much lower than the tensile strength of 210.8 MPa. All four of the verified elements were able to withstand the loads. Therefore, only the SP and SP elements were taken for further analysis due to their much lighter weights, which would benefit the handling of the elements.

## 6. Numerical Modeling of an Acoustic Screen

**Figure 12:**
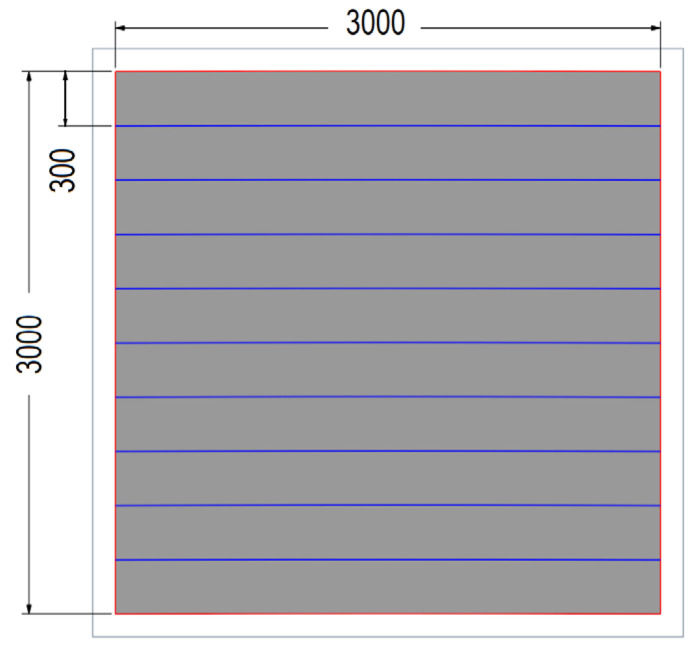
Road screen acoustic panel.

**Figure 13:**
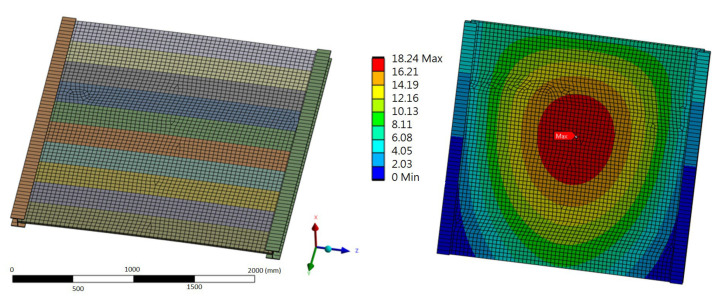
Division into finite elements and coordinate system and panel deflection values (mm).

**Figure 14:**
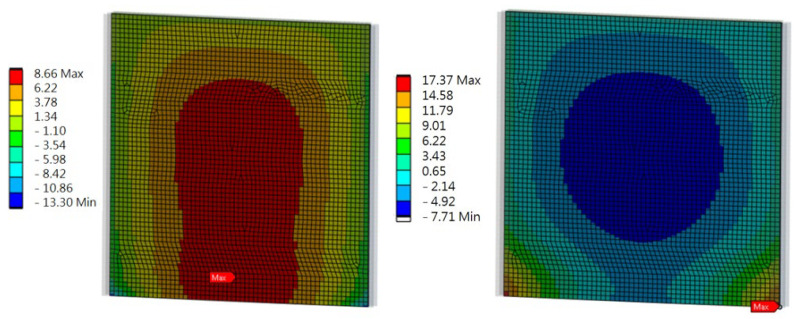
Stresses on the leeward and windward sides of the panel in the Z direction (MPa).

**Figure 15:**
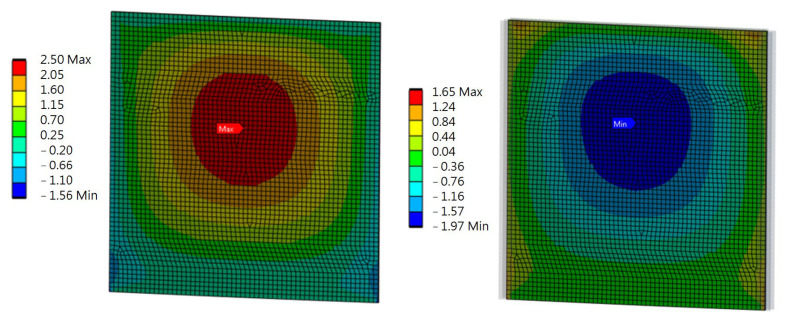
Stresses on the leeward and windward sides of the panel in the Y direction (MPa).

Ten rectangular components, each measuring 3000 mm by 300 mm, which were cut from wind turbine blades, as illustrated in Figure 6, Figure 7 and Figure 8, made up the road screen acoustic panel. These elements were made exclusively from D-type laminate, as it demonstrated sufficient load resistance with a much lower weight. The material model of the columns and frame was adopted as being typical for steel, while the material model of the panel was taken from experimental tests of the D-type laminate (sandwich). The connection of the composite elements of the panel with the stiffening frame was modeled as flexible. The elements of the frame were modeled as 1D beam elements, while 2D shell elements constituted the panel. The size of the rectangular 2D finite element was 5 cm × 5 cm. The results of the analysis of the acoustic panel are displayed in Figure 12, Figure 13, Figure 14 and Figure 15.

Numerical modeling of an acoustic screen showed that the largest stress values in the panel under loading were 17.4 MPa (tensile stress) and 13.3 MPa (compressive stress) in the Z direction. The largest stress values in the Y direction were 2.5 MPa (tensile stress) and 2.0 MPa (compressive stress). The places where the highest stress values occurred were the center of the panel and the sides, where the panel was fixed to the frame.

## 7. Panel Failure Analysis

To check the possibility of panel failure as well as verify its load-bearing capacity, the ultimate limit states (ULS) method for verification contained in [18] was used. The ULS approach provides a relatively simple and consistent framework for structural analysis and design. This is a major advantage when using composite materials, where there are various failure criteria, depending on the type of material used and the loading conditions. Moreover, in many European jurisdictions, compliance with Eurocodes or similar standards is mandatory for structural design. Adhering to the ULS approach helps engineers meet legal and regulatory requirements and ensures that structures comply with safety standards.

Only the ULS conditions regarding the face sheets of sandwich profiles were verified. The conditions were as follows: face sheet tensile failure, face sheet crushing, and face sheet wrinkling. To conduct the verification, the maximum stresses shown in Figure 14 and Figure 15 were used.

The face sheet tensile failure (Z direction) is calculated as follows:

(7) $${\left(\sigma_{z,Ed}\right)}_{f}\leq {\left(f_{z,t,d}\right)}_{f}$$

where ${\left(\sigma_{z,Ed}\right)}_{f}$ is the design value of the in-plane tensile stress in the face sheets, ${\left(\sigma_{z,Ed}\right)}_{f}=17.4\;MPa$, ${\left(f_{z,t,d}\right)}_{f}$ is the design value of the in-plane tensile strength in the Z direction, and ${\left(f_{z,t,d}\right)}_{f}=21.2\;MPa$, and thus

17.4 MPa≤21.2 MPa.

The face sheet tensile failure in the Y direction is expressed as

(8) $${\left(\sigma_{y,Ed}\right)}_{f}\leq {\left(f_{y,t,d}\right)}_{f}$$

2.5 MPa≤15.6 MPa.

Therefore, the condition was passed.

The face sheet crushing in the Z direction is expressed as

(9) $${\left(\sigma_{z,Ed}\right)}_{f}\geq {\left(f_{z,c,d}\right)}_{f}$$

where ${\left(\sigma_{z,Ed}\right)}_{f}$ is the design value of the in-plane compressive stress in the face sheets, ${\left(\sigma_{z,Ed}\right)}_{f}=-13.3\;MPa$, ${\left(f_{z,c,d}\right)}_{f}$ is the design value of the in-plane compressive strength in the Z direction, and ${\left(f_{z,c,d}\right)}_{f}=-27.3\;MPa$, and thus

−13.3 MPa≥−27.3 MPa.

The condition was passed.

The face sheet crushing in the Y direction is calculated as follows:

(10) $${\left(\sigma_{y,Ed}\right)}_{f}\geq {\left(f_{y,c,d}\right)}_{f}$$

−1.96 MPa≥−40.1 MPa.

The condition was passed.

The face sheet wrinkling in the Z direction is expressed as

(11) $$\left|{\left(\sigma_{z,Ed}\right)}_{f}\right|\leq {\left(f_{z,wr,d}\right)}_{f},$$

where ${\left(\sigma_{z,Ed}\right)}_{f}$ is the design value of the in-plane compressive stress in the face sheets, ${\left(\sigma_{z,Ed}\right)}_{f}=17.4\;MPa$, and ${\left(f_{z,wr,d}\right)}_{f}$ is the design value of the wrinkling stress in the Z direction such that

(12) $${\left(f_{z,wr,d}\right)}_{f}=\frac{1}{\gamma_{m}\times \gamma_{Rd}}\cdot {\left(f_{z,wr,k}\right)}_{f}$$

(13) $${\left(f_{z,wr,k}\right)}_{f}=0.65\cdot \sqrt[{3}]{\left[{\left(\eta_{c}\right)}_{f}\cdot {\left(E_{z,c,k}\right)}_{f}\right]\cdot \left[{\left(\eta_{c}\right)}_{c}\cdot {\left(E_{z,k}\right)}_{f}\right]\cdot \left[{\left(\eta_{c}\right)}_{c}\cdot {\left(G_{xz,k}\right)}_{c}\right]}$$

where ${\left(\eta_{c}\right)}_{f}$ is the conversion factor of the face sheet, ${\left(\eta_{c}\right)}_{c}$ is the conversion factor of the core, ${\left(E_{z,c,k}\right)}_{f}$ is the characteristic value of the compressive modulus in the Z direction of the face sheet, ${\left(E_{z,k}\right)}_{f}$ is the characteristic value of the out-of-plane elastic modulus of the core, and ${\left(G_{xz,k}\right)}_{c}$ is the characteristic value of the out-of-plane shear modulus (XZ plane) of the core such that

(14) $${\left(\eta_{c}\right)}_{f}={\left(\eta_{ct}\right)}_{f}\cdot {\left(\eta_{cm}\right)}_{f}$$

$${\left(\eta_{c}\right)}_{f}=0.93\cdot 0.85=0.79$$

(15) $${\left(\eta_{c}\right)}_{c}={\left(\eta_{ct}\right)}_{c}\cdot {\left(\eta_{cm}\right)}_{c}$$

$$\eta_{c}=0.99\cdot 0.85=0.85$$

(16) $${\left(f_{z,wr,k}\right)}_{f}=0.65\cdot \sqrt[{3}]{\left[0.79\cdot 29.3\;GPa\right]\cdot \left[0.85\cdot 3\;GPa\right]\cdot \left[0.85\cdot 0.3\;GPa\right]}=0.94\;GPa$$

$${\left(f_{z,wr,d}\right)}_{f}=\frac{1}{1.3\cdot 1.5}\cdot 0.94\;GPa=482\;MPa$$

$$\left|-13.3\;MPa\right|\leq 482\;MPa$$

The condition was passed.

The face sheet wrinkling in the Y direction can be expressed as

(17) $$\left|{\left(\sigma_{y,Ed}\right)}_{f}\right|\leq {\left(f_{y,wr,d}\right)}_{f}$$

where

$${\left(f_{y,wr,k}\right)}_{f}=0.65\cdot \sqrt[{3}]{\left[0.79\cdot 6.5\;GPa\right]\cdot \left[0.85\cdot 0.05\;GPa\right]\cdot \left[0.85\cdot 0.3\;GPa\right]}=0.248\;GPa$$

(18) $${\left(f_{y,wr,d}\right)}_{f}=\frac{1}{\gamma_{m}\cdot \gamma_{Rd}}{\left(f_{y,wr,k}\right)}_{f}=\frac{1}{1.3\cdot 1.5}0.248\;GPa=127\;MPa$$

$$\left|-1.96\;MPa\right|\leq 127\;MPa.$$

All of the required ULS conditions were met. This indicates that, according to the standard [18], the panel is able to withstand the necessary loads.

## 8. Acoustic Screens’ Effects on the Environment

The environment is greatly impacted when road noise barriers are built. At the moment, tempered glass, acrylic, wood, metal (metal plates packed with sound-absorbing material, like glass wool), and concrete are the materials used to make acoustic screens most frequently. A composite made from wind turbine blades can be used in place of these virgin raw materials. This can be reused with much less effort than recycling and has more potential environmental benefits [19]. Using the life cycle assessment (LCA) method, sound barriers’ environmental impact can be evaluated by considering how a product interacts with the environment at each stage of its life, from raw material production to recycling or scrapping. This impact can be minimized by reducing the impact of the material used to make the barriers, but this method ignores the procedures involved in the actual manufacturing, shipping, and installation of the barrier.

For the purpose of this article, sound barriers composed of composite material recovered from wind turbine blades may be evaluated for their potential to reduce environmental impact by using these values as a starting point. For a single slab of completed barrier constructed from each material, Table 6 displays the embodied values (cradle-to-gate range) of their energy consumption and greenhouse gas emissions [20].

It should be mentioned that the composite material used to produce sound barriers made from wind turbine blades is a reusable waste that can be used instead of virgin raw materials. After accounting for procedures like cutting wind turbine blades, fabricating steel poles, and transporting and installing the sound barrier, the environmental impacts of using waste materials instead of virgin ones should be viewed as negative and eventually deducted from the overall environmental impact of the resulting sound barrier. The benefits of material substitution can be effectively measured in this situation using the LCA method. The authors plan to analyze these benefits at a later stage in their research.

## 9. Acoustic Characteristics of Composite Sandwich Components Filled with Balsa

Numerous research works have looked into the integrated sandwich structure of natural materials and how well they absorb sound. It was found that the multiscale structure of natural materials was responsible for their remarkable sound energy absorption performance and intricate energy dissipation mechanism [21]. The multi-layer sandwich structure allows for a more effective and gradual dissipation of sound energy when compared with individual core materials or composite laminates, especially at high frequencies. With the right design, the sandwich structure composed of natural materials can be used as a multifunctional, load-bearing, and sound-absorbing structure, particularly at high frequencies. Because of the weight restrictions and the high-frequency service environment, this would be quite helpful in road construction for the construction of noise barriers to protect residential areas from noise. The experimental results show that the use of natural fiber composite face sheets with a balsa core can double the coincidence frequency in a sandwich composite construction, and the use of composite fiber face sheets with a synthetic core can more than triple it [22]. Furthermore, sandwich composites with better acoustic performance are correlated with core materials having a low specific shear modulus. The amount of noise radiation, as shown by the wave number amplitudes, was significantly reduced when employing natural materials, even though the damping values of the sandwich composites based on natural materials are still comparable to those of conventional synthetic sandwich composite beams. The use of natural materials in sandwich composite materials, which are renewable, recyclable, and biodegradable, thus holds promise for the development of environmentally friendly materials and for solving the issue of noise radiation from sandwich structures.

High-porosity wood has a small specific impedance, which makes it an effective sound-absorbing material, according to Smardzewski et al. [23]. They found that in boards made of balsa or binuang, the higher the sound absorption coefficient, the lower the density. This is due to the fact that higher pore contents result in lower densities, which causes more sound waves to be reflected and dispersed, losing energy.

All of these studies came to the same conclusion: because the glass fiber-balsa composite has adequate acoustic qualities, it can be a good material choice for roadside noise barriers.

## 10. Conclusions

One of the methods for achieving sustainable development is to implement a circular economy, in which extending the life of a product is achieved by using waste as raw materials to create other products. In line with this strategy, the aim of the article was to propose a method of using waste in the form of unusable wind turbine propellers as materials for the construction of road noise barriers. The first part of the article discussed the construction of noise barriers and the principles of their design, and it proposed the construction of a screen in which the acoustic panel is made of composite elements obtained from wind turbine propellers. Then, the loads acting on the screen were collected in accordance with the requirements of the European standards, and the mechanical properties of the screen’s composite material were determined.

The next step was to build numerical models of the composite elements constituting the screen and check their resistance to loads related to wind pressure and the impact of car traffic. For this purpose, two types of composite elements, solid and sandwich, were cut from the propeller blade structure, and their behavior under the design load was determined. The comparison showed that the greatest utilization of the load-bearing capacity of a sandwich element in bending was 16.8%.

Then, a numerical model of the entire acoustic screen was developed, consisting of a composite acoustic panel fitted between load-bearing columns made of HEA160 I-beams. The material model for the panel was derived from D-type laminate (sandwich) experimental tests, whereas the material model for the columns was adopted based on typical steel strength values. The design loads required by the standard [9] were applied to the model. The maximum stresses in the main directions (Y and Z) due to the applied loads were determined through the use of finite element analysis.

The largest stress value in the panel under loading, according to numerical modeling of an acoustic screen, was 17.4 MPa for the tensile stress and 13.3 MPa for the compressive stress in the Z direction. The tensile stress (2.5 MPa) and compressive stress (2.0 MPa) were the two largest stress values in the Y direction. The panel’s center and the sides, where the panel was fastened to the frame, had the highest stress values.

These values were used to verify the panel’s mechanical performance and determine the possibility of its failure. For the verification, the ULS conditions for sandwich composites contained in [18] were used. The verification showed that the acoustic screen panel was able to withstand the loads without face sheet failure.

The use of composite material made from recycled wind turbine blades to make acoustic screens has significant environmental effects in the form of minimizing energy consumption and CO_2_ emissions, as shown in the outcomes of the life cycle analysis for various types of acoustic screens in Table 6.

### Research Currently Underway

**Figure 16:**
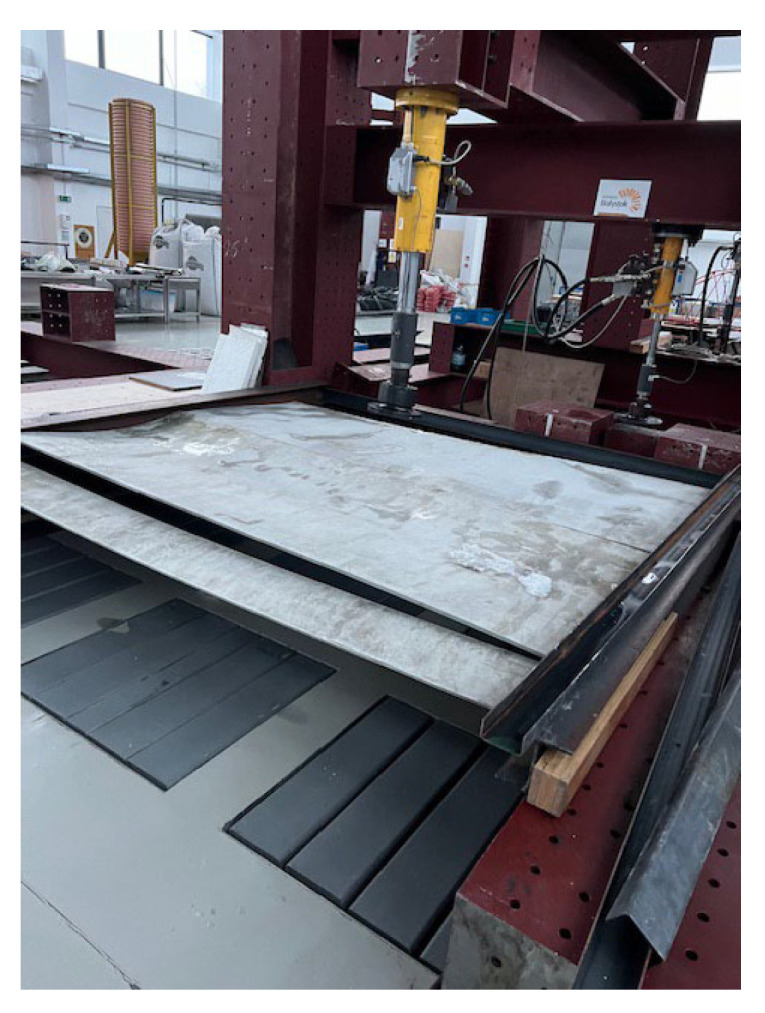
Acoustic panel mounted on a research stand.

At the moment, natural-scale experimental testing of acoustic panels is being conducted on a research stand (Figure 16). The elements are embedded in steel columns with an HEA 160 cross-section. Care is taken to fit the flat components of the acoustic panel quite precisely in order to guarantee continuous contact between them. Strong acrylic tapes that fit well on the joined surfaces are being used to connect the cut-out elements on both sides. The tests are designed to ascertain the acoustic panel’s load-bearing capacity and the degree of deformation caused by dynamic loads from snow removal and wind pressure. The adhesive joints’ susceptibility will additionally be verified during testing. A comparison with the panel performance during the finite element analysis will also be conducted to confirm the FEA results.

## Figures and Tables

**Table 1:** Values of the mechanical and physical properties of individual types of laminate [11].

Properties	Laminate C	Laminate D	
		Outer Layers ^(1)^	Balsa ^(2)^
Characteristic Values			
Thickness *t* (mm)	29.6	3.0	24.0
Young’s modulus in the Z direction *E_z_* (GPa)	24.8	5.9	(0.61–6.6)
Young’s modulus in the Y direction *E_y_* (GPa)	6.4	6.5	(0.013–0.098)
Kirchhoff module *G_xz_* (GPa)	2.7	3.1	(0.04–0.36)
Own weight ρ (kG/m^3^)	1976	1976	163
Compressive strength in the Z direction Z*_Ck_*(MPa)	344.2	217.7	-
Compressive strength in the Y direction *Y_Ck_* (MPa)	145.8	159.7	-
Tensile strength in the Z direction Z*_Tk_*(MPa)	405.8	88.5	-
Tensile strength in the Y direction *Y_Tk_* (MPa)	52.8	119.6	-
Shear strength *S_xy_* (MPa)	26.4	44.2	(1.3–3.1)
ULS Design Values			
Compressive strength in the Z direction *Z_Cd_* (MPa)	183.8	27.3	
Compressive strength in the Y direction *Y_Cd_* (MPa)	59.5	40.1	
Tensile strength in the Z direction *Z_Td_* (MPa)	210.8	21.2	
Tensile strength in the Y direction *Y_Td_* (MPa)	25.1	15.6	
Shear strength *S_xy_* (MPa)	12.6	10.6	(1.5–3.5)

^(1)^ Averaged values from the outer and inner layers obtained from the tests [11,12]. ^(2)^ Values obtained from the literature [14].

**Table 2:** Layup of the laminates.

Name	C-Type Laminate	D-Type Laminate
Type	Solid	Sandwich
Thickness	30mm	3 mm outer layer 24 mm core balsa layer 3 mm inner layer

**Table 3:** Dimensions, types, and weights of the elements.

Element Name	Length (mm)	Thickness (mm)	Height (mm)	Volume (cm^3^)	Unit Weight (kg/m^3^)	Weight (kg)
GL (L) ^(1)^	3000	30	300	27,003	1976	53.4
GP (L)	3000	30	300	27,003	1976	53.4
SL (S)	3000	30	300	27,015	525	14.2
SP (S)	3000	30	300	27,087	525	14.2
DL (S)	3000	30	300	27,004	525	14.2
DP (S)	3000	30	300	27,011	525	14.2

^(1)^ Type of laminate. (L) = solid; (S) = sandwich.

**Table 4:** Design variable load values.

Wind load	$w_{e,d}=1126\;N/m^{2}$
Traffic load	$q_{d}\left(v\right)=1200\;N/m^{2}$

**Table 5:** Verification of strength for solid elements GP and GL and sandwich elements SP and SL.

**Type of Stress (MPa)**	**Resistance Condition**
GP element	
Compression when bending about the Y axis	$\sigma_{y.c}=-4.1\;MPa<Z_{Cd}=183.8\;MPa$
Tension when bending about the Y axis	$\sigma_{y.t}=5.4\;MPa<Z_{Td}=210.8\;MPa$
Compression when bending about the Z axis	$\sigma_{z.c}=-1.2\;MPa<Y_{Cd}=59.5\;MPa$
Tension when bending about the Z axis	$\sigma_{z.t}=1.7\;MPa<Y_{Td}=25.1\;MPa$
GL element	
Compression when bending about the Y axis	$\sigma_{y.c}=-5.3\;MPa<Z_{Cd}=184.8\;MPa$
Tension when bending about the Y axis	$\sigma_{y.t}=4.3\;MPa<Z_{Td}=210.8\;MPa$
Compression when bending about the Z axis	$\sigma_{z.c}=-1.6\;MPa<Y_{Cd}=59.5\;MPa$
Tension when bending about the Z axis	$\sigma_{z.t}=1.3\;MPa<Y_{Td}=25.1\;MPa$
SP element	
Compression when bending about the Y axis	$\sigma_{y.c}=4.6\;MPa<Z_{Cd}=27.3\;MPa$
Tension when bending about the Y axis	$\sigma_{y.t}=2.9\;MPa<Z_{Td}=21.2\;MPa$
Compression when bending about the Z axis	$\sigma_{z.c}=0.2\;MPa<Y_{Cd}=40.1\;MPa$
Tension when bending about the Z axis	$\sigma_{z.t}=0.4\;MPa<Y_{Td}=15.6\;MPa$
SL element	
Compression when bending about the Y axis	$\sigma_{y.c}=4.5\;MPa<Z_{Cd}=27.3\;MPa$
Tension when bending about the Y axis	$\sigma_{y.t}=3.0\;MPa<Z_{Td}=21.2\;MPa$
Compression when bending about the Z axis	$\sigma_{z.c}=0.4\;MPa<Y_{Cd}=40.1\;MPa$
Tension when bending about the Z axis	$\sigma_{z.t}=0.2\;MPa<Y_{Td}=15.6\;MPa$

**Table 6:** The embodied values (cradle-to-gate range) of energy consumption and greenhouse gas emissions for one slab of finished barrier [20].

Barrier Type	Materials	Mass Per Unit Plate (kg)	Embodied Energy (MJ)	Embodied Carbon (kgCO_2eq._)
Acrylic sound barrier	Acrylic board/aluminium frame	24/3.33	2693.01	22.24
Metal composite sound barrier board	Galvanized steel/glass wool/aluminium frame	12.56/32/4.28	1842.48	115.53
Wooden sound barrier	Wood	150	2400.00	122.25
Concrete sound barrier	Concrete/glass fiber	150/32	1019.00	60.45
Fly ash cenosphere cement-based sound barrier board	Cement/fly ash cenosphere/glass fiber/metal frame	64/36/20/4.28	477.08	57.57

## Data Availability

Data are contained within the article.
